# Laparoscopic partial pancreatectomy through an advanced lateral approach as treatment for insulinoma in dogs: a case series

**DOI:** 10.3389/fvets.2023.1278218

**Published:** 2024-01-08

**Authors:** Justin N. P. Keulen, Sebastiaan A. van Nimwegen

**Affiliations:** Department of Clinical Sciences of Companion Animals, Faculty of Veterinary Medicine, Utrecht University, Utrecht, Netherlands

**Keywords:** abdominal surgery, minimally invasive, pancreas, neoplasia, endocrine, neuroendocrine, companion animals, canine

## Abstract

A minimally invasive partial pancreatectomy was performed through a novel laparoscopic lateral flank approach in sternal-oblique recumbency in four clinical cases presented with an insulinoma. All four cases were female castrated dogs of older age (range 7–9 years) and different breeds (Wire haired dachshund, German shepherd, Jack Russel terrier, and Boxer), and all presented with episodic weakness, tremors, and/or seizures. The diagnosis was based on plasma glucose level below reference range with concomitant increased or normal insulin level. A laparoscopic approach was considered based on triple-phase contrast-enhanced computed tomography findings of the abdomen, revealing a pancreatic mass situated in the right pancreatic limb or left pancreatic limb without suspicion of metastasis. Laparoscopic procedures were performed without any major complications, and peri-operative glycemia increased to (supra-)normal levels in all cases. Histopathologic reports qualified the masses as neuroendocrine carcinomas, and in conjunction with the clinical picture, this neoplasia was further defined as insulinoma. Post-operative care in an intensive care unit was of short duration, and all animals were discharged being clinically normal and normoglycemic in between 1.5 and 2.5-day post-surgery. At short-term follow-up, no dogs showed clinical abnormalities, all recovered well from the surgical procedure, and blood glucose levels remained in the normal range. During long-term follow-up, 2 cases remained clinically normal at the time of writing, 564 and 1,211 days after surgery, 1 dog had recurrence of hypoglycemic episodes after 246 days and was euthanized after 673 days of surgery due to progressing disease, and 1 dog was euthanized after 1,028 days of surgery due to reasons unrelated to the insulinoma. Survival times ranged from 599 to 1,232 days after diagnosis. Considering the highly metastatic nature and difficulty of full laparoscopic staging of insulinoma patients, thorough pre-operative disease staging is warranted when considering a laparoscopic approach. This case series shows the feasibility of a novel laparoscopic flank approach for right and left partial pancreatectomy in dogs. Furthermore, proper case selection resulted in favorable outcome in these insulinoma patients.

## 1 Introduction

Insulinomas are an endocrine neoplasia of the pancreas of dogs. They arise from pancreatic β cells in the islets of Langerhans and cause hypoglycemia and its symptoms by hypersecretion of insulin (1). While these functional neoplasias are more frequently observed in medium to large breed dogs, they also do occur in smaller dogs. Additionally, dogs of older age are mostly presented (2, 3). Insulinomas are regarded as malignant neoplasms in dogs because of the high rate of metastasis, primarily to the abdominal lymph nodes and liver (4).

Clinical symptoms of neuroglycopenia are most commonly observed, including seizures, weakness, posterior paresis, collapse, and proprioceptive ataxia, often occurring in an episodic manner. Other common symptoms include muscle fasciculation, nervousness, and polyphagia. Physical examination is unremarkable in most dogs (3–5).

Primary diagnosis is made by assessing blood glucose levels in conjunction with blood insulin levels at the time of clinical signs or after a fasting period. Hypoglycemia, paired with normoinsulinemia or hyperinsulinemia, is characteristic for insulinoma (1, 2, 4). In conjunction with blood tests, medical imaging is regularly performed to assess the pancreas for mass-like lesions and check for locoregional or distant metastasis. Abdominal ultrasound with or without contrast enhancement (6), magnetic resonance imaging (MRI) (7), radiography (2), and scintigraphy (8) has been used to detect a pancreatic mass lesion, define its location, and assess for metastasis. Contrast-enhanced computed tomography (CECT) is, however, superior to other modalities and can accurately detect insulinomas and visualize potential metastatic lymph nodes. Triple-phase CECT is of superior value over dual-phase assessment because the degree of contrast enhancement is unpredictable and varies per tumor and per contrast angiography phase. Therefore, multiphase CECT increases diagnostic value for insulinoma detection (9–11). Presentation on CECT can differ, insulinomas can either by hypo- or hyperattenuating, homo- or heterogeneously enhancing, and well- or ill-defined, depending on phase of contrast enhancement (12). Primary insulinomas usually occur as a solitary, mass-like lesion anywhere in the pancreas, often of relatively small size (1). They can occasionally be found more diffusely distributed in the pancreas (5). Potential metastatic lymph nodes or liver lesions can be diagnosed by ultrasound, CECT, and intraoperative inspection and palpation, and metastasis is found to be present in approximately half of the insulinoma cases at first presentation (3).

Insulinomas can be treated medically and surgically. Medical management of sequalae of insulinomas consists of treatment of hypoglycemia, suppression of insulin blood levels, and dietary measures (1). Chemotherapeutic treatment is of limited value in canine insulinoma; however, tyrosine kinase inhibitors have shown some promising results (1, 13). Surgical treatment, if possible, is the treatment of choice, with higher median survival times (MST) compared with other treatment options (5).

Prognosis, in general, is guarded, however, the prognosis is better for dogs that are (initially) surgically treated compared with medical therapy alone. Other positive prognostic indicators are normoglycemia or hyperglycemia after surgical treatment, small tumor size and low clinical stage of disease, and a Ki-67 index ≤2.5% (3–5, 14, 15). Specific complications after insulinoma surgery include diabetes mellitus and pancreatitis, which warrant proper postoperative patient monitoring and prompt medical treatment if indicated (5).

**Figure 1:**
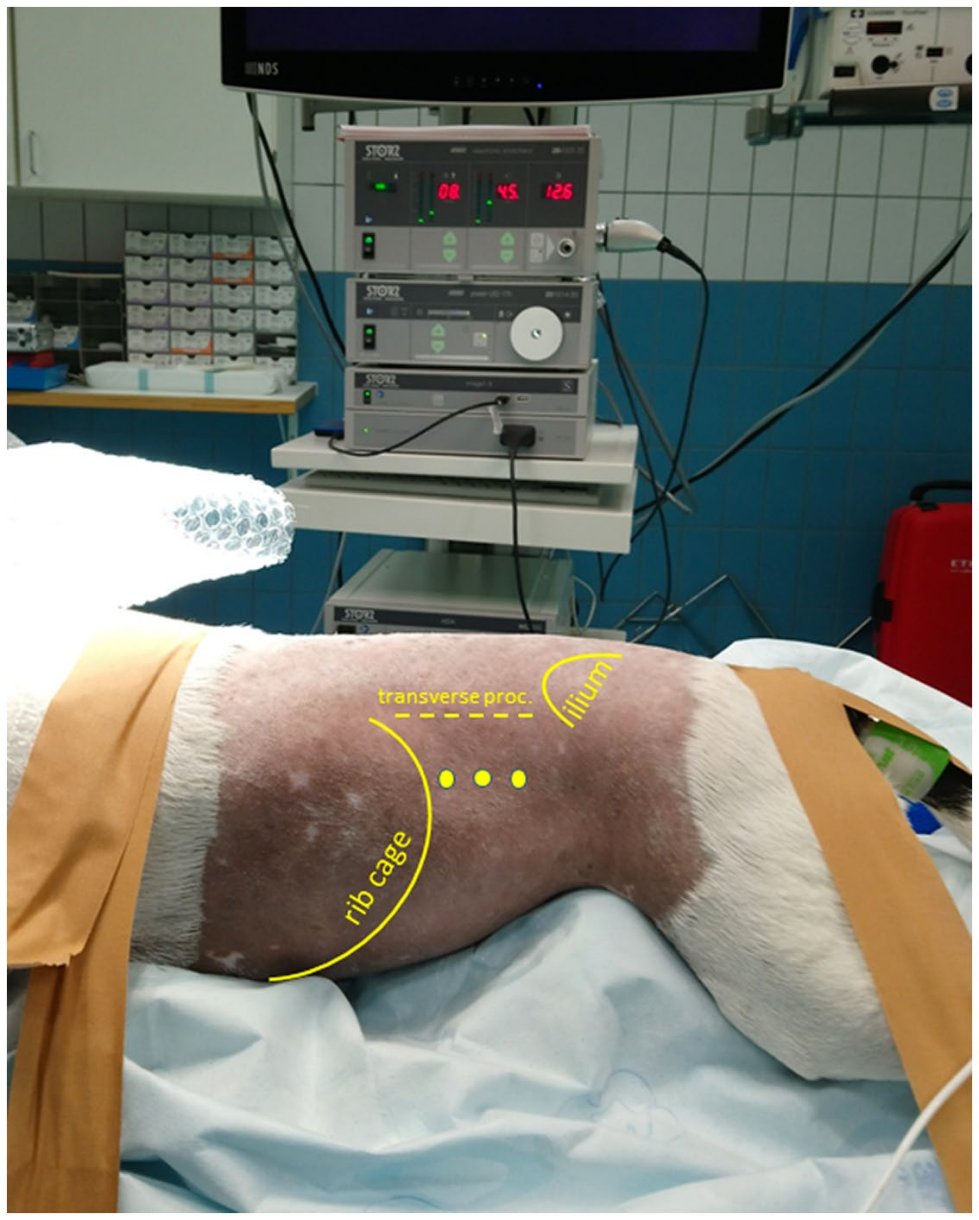
Patient positioned in sternal recumbency and portal placement for a (left) laparoscopic flank approach. Positioning of the patient in sternal, slightly right lateral oblique recumbency with elevation of thorax and pelvis. The initial camera portal is placed 1–3 cm caudal of the last rib and 3–5 cm ventral of the vertebral transverse processes. The location of two additional portals for a triangulated surgical approach depends on the intraoperative situation.

Insulinomas are preferably excised by partial pancreatectomy, aiming for a margin of tumor-free tissue. Local enucleation, or marginal excision, may be performed if anatomy does not permit wider margins, such as tumors located in the corpus or near a pancreatic duct. Bipolar vessel-sealing devices improve marginal dissections and probably reduce complications compared with other partial pancreatectomy techniques because blunt pancreatic dissection is greatly reduced, and pancreatic tissue is sealed upon transection (16). Laparoscopic approaches in pancreatic surgery have been well documented in human medicine and seem to have advantages over an open approach (17–19). One case report described laparoscopic excision of an insulinoma in the left pancreatic lobe in a dog through a ventral abdominal approach with the dog in dorsal recumbency (20). In author’s experience, however, laparoscopic access to the left or right pancreatic lobe is greatly improved through a left or right lateral flank approach, respectively, with the patient in sternal recumbency, as an approach was previously described for adrenalectomy in dogs (21). By positioning the animal in sternal recumbency (Figure 1), gravitation causes displacement of abdominal organs ventrally, and hence, better visibility and manipulation opportunities of structures located more dorsally in the abdomen. Because of the high metastatic rate of canine insulinomas and the difficulty in complete lymph node staging through a laparoscopic approach, proper disease staging using multi-phase CECT is considered mandatory in these cases. Furthermore, blood glucose levels should be monitored intraoperatively since an immediate increase from blood glucose levels to physiologic or supraphysiologic levels, after tumor excision, is expected in cases without measurable metastasis. If glucose levels do not increase significantly, further surgical exploration and excision of possible metastatic lymph nodes should be considered. The present case report describes the diagnostic work-up, surgical approach, surgical procedure, and immediate-, short-, and long-term follow-up of four clinical cases with an insulinoma of the pancreatic limb treated by partial pancreatectomy through this innovative laparoscopic flank approach.

## 2 Methods

### 2.1 Surgical anatomy

**Figure 2:**
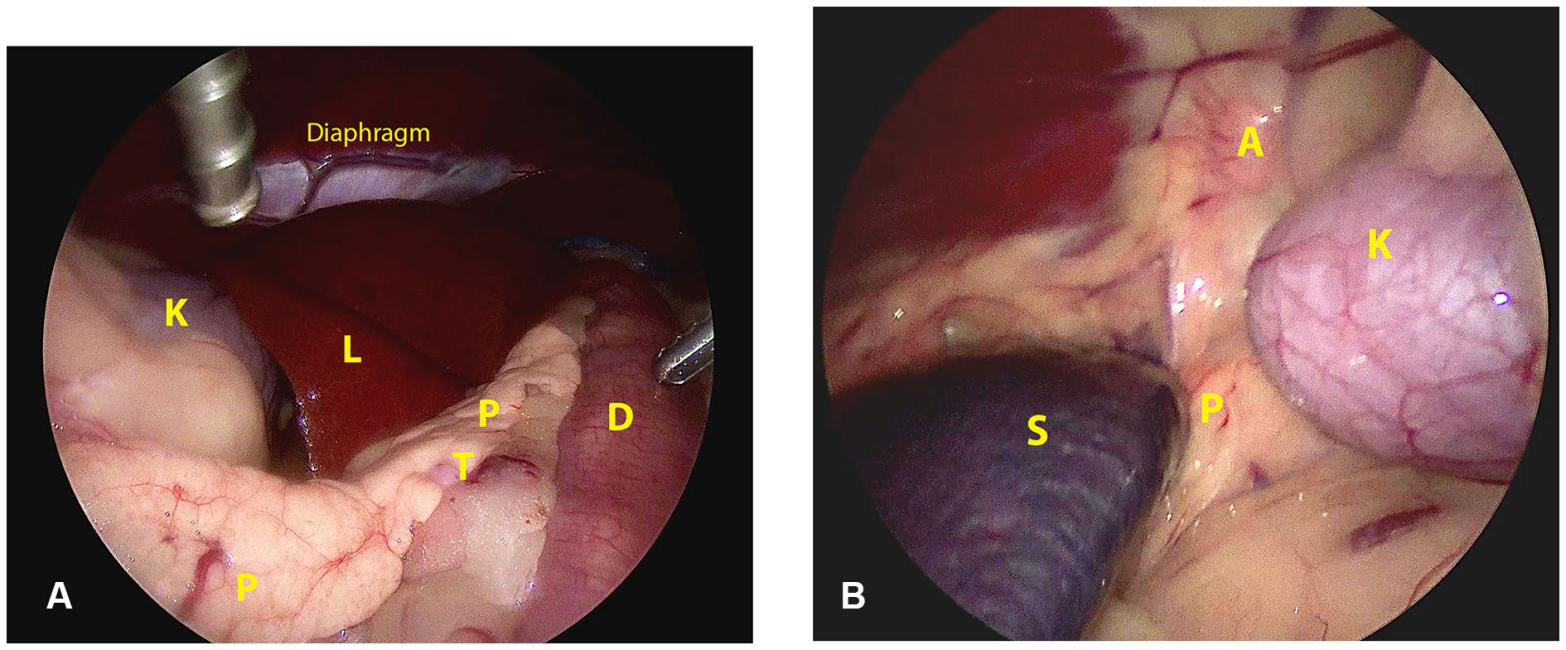
Surgical view and anatomic references directly after abdominal entry through a right **(A)** and left **(B)** lateral laparoscopic approach in sternal recumbency. A: adrenal gland; D: duodenum; K: kidney; L: liver; P: pancreas; S: spleen; T: tumor.

**Figure 3:**
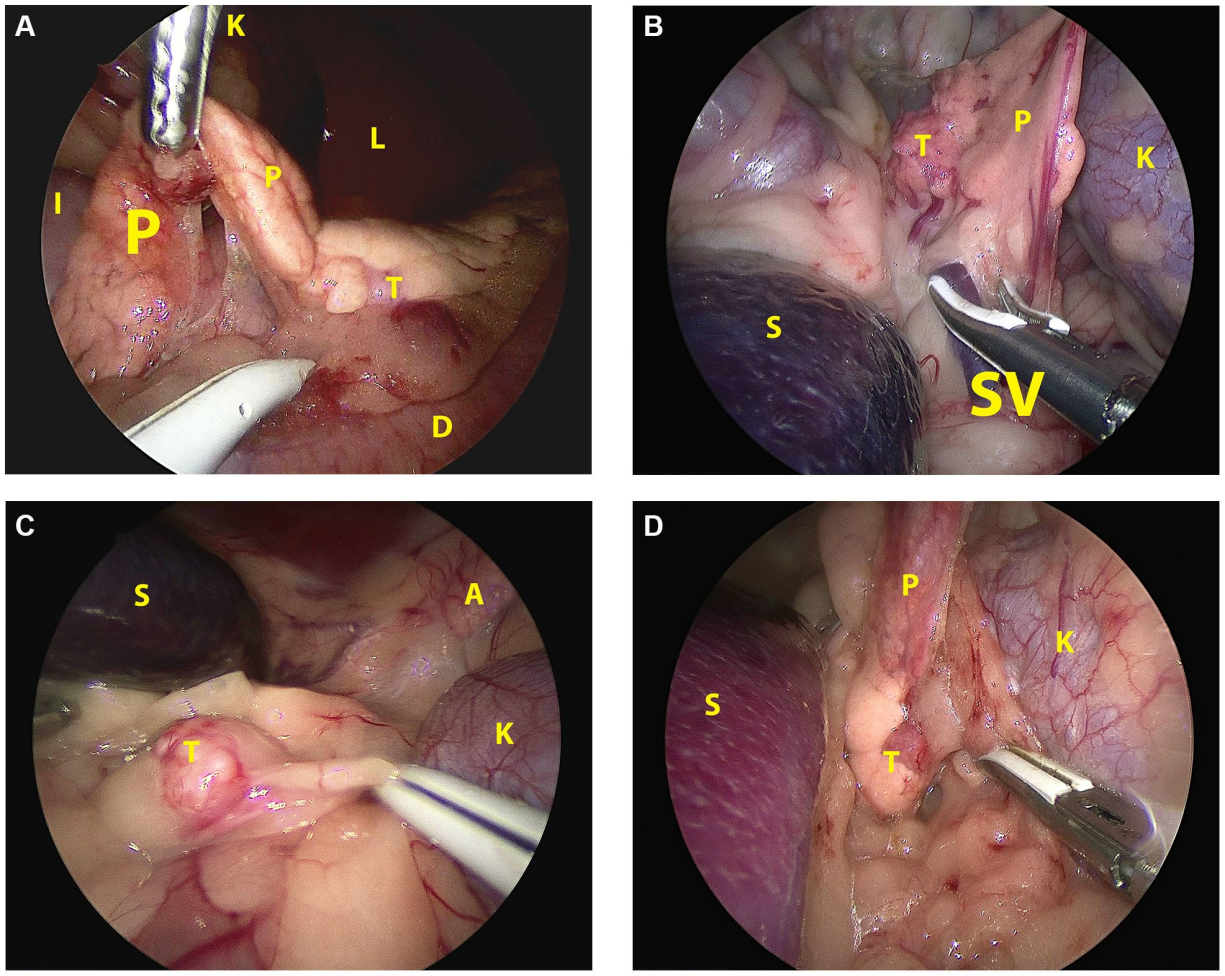
Laparoscopic image of the insulinoma situated in the affected pancreatic lobe during pancreatectomy in sternal recumbency in each patient. **(A)**: Case 1, right flank approach, mid right lobe; **(B)**: case 2, left flank approach, mid left lobe; **(C)**: case 3, left flank approach, distal left lobe; **(D)**: case 4, left flank approach, proximal left lobe. A: adrenal gland; D: duodenum; I: ileum; K: kidney; L: liver; P: pancreas; S: spleen; SV: splenic vein; T: tumor.

The topographic and surgical anatomy of the pancreas and surrounding organs in sternal recumbency with ‘hanging belly’ was previously evaluated in a cadaver model (22). Briefly, in a right flank approach, the lateral surface of the complete right pancreatic limb lies in plain sight with the duodenum situated ventrally by gravity (Figure 2A). The pancreatic corpus (dorsal surface) may be obscured by the liver but can be approached by following the duodenum and right pancreatic limb cranially while retracting the right middle liver lobe cranially. This may also reveal the proximal part of the left pancreatic lobe. The medial side of the right pancreatic lobe can be visualized by deflecting the duodenum dorsally (22). In the left flank approach, the dorsal surface of the tip of the left pancreatic lobe may be visible as it is situated in the dorsal leaf of the greater omentum, craniomedioventral to the left kidney, dorsomedial to the spleen (Figure 2B). However, often the left lobe is not readily visible because it is obscured by the splenic part of the omentum that runs toward the mesocolon, which may also contain a considerable amount of fat, further hindering visibility, especially in insulinoma patients. The dorsal surface of the left pancreatic lobe can be visualized by pulling the omentum cranially. Once the omental sac is opened over the area of the pancreas, the ventral side of the tip of the left limb is readily accessible, and minor traction easily reveals a larger part of the left limb (Figures 3B–D). A combination of traction on the left pancreatic limb and dissection of its attachment to the dorsal omental leaf reveals a larger part of the limb, and dissection can be continued toward the corpus.

### 2.2 Inclusion

Dogs with insulinoma referred to our tertiary care University Animal Hospital, department of Clinical Sciences, faculty of Veterinary Medicine, Utrecht University, the Netherlands, between 01-01-2019 and 01-01-2022 that met the following inclusion criteria were considered for laparoscopic treatment: Complete diagnostic work-up, including plasma glucose and insulin levels, triple-phase CECT staging of the abdomen, and CT evaluation of the thorax. Additional ultrasound-guided fine needle biopsies of any abnormal lymph node or liver lesion were performed to exclude possible metastasis. If a solitary nodule was detected in a pancreatic lobe without signs of metastasis in abdominal lymph nodes or thorax on CECT and no indication of metastasis was observed on imaging or cytology of the liver, patients were scheduled for laparoscopic surgery with the option of conversion to an open approach if indicated. In the case of suspicious lymph nodes or other signs of metastasis on CECT and/or tumors located in or close to the pancreatic corpus, or other possible complicating factors, an open surgical approach through ventral midline celiotomy was advised.

### 2.3 Surgical approach

The surgeries were performed by a certified diplomate of the European College of Veterinary Surgeons with considerable experience in advanced minimally invasive surgical procedures. Patients were positioned in sternal recumbency with the thorax and pelvis elevated and stabilized by a moldable vacuum cushion ensuring that the abdomen could freely hang down to promote the abdominal organs to move ventrally (Figure 1). Depending on which flank was approached surgically, the patient was positioned up to 30^o^ obliquely toward the contralateral side. In the case of a planned or anticipated bilateral approach, the patient was placed in complete sternal recumbency ensuring that both flanks were approachable for surgery without re-draping. Apart from the carefully shaped moldable vacuum cushions, patients were also fixated with tape or cotton bands to the motorized tiltable operating table that facilitated another 30^o^, further tilting in all directions if indicated (based on intra-abdominal situation during surgery). The initial portal was placed 1–3 cm caudal of the last rib and 3–5 cm ventral of the vertebral transverse process through an open modified Ternamian entry technique using a threaded cannula with an outer diameter of 6 mm (Ternamian endotip; Karl-Storz-Endoscopy, Amersfoort, the Netherlands) and a laparoscope with a diameter of 5 mm with 30° viewing angle (Hopkins® II, Karl Storz Endoscopy). The laparoscope was connected to a 4 K/UHD video camera system (Storz Image 1S 4U, Karl Storz Endoscopy), with recording option (23). After visual confirmation of abdominal entry, the abdomen was insufflated with CO_2_ to an intra-abdominal pressure of 8 mmHg (CO2 Endo-ArthroflatorTM-Vet, Karl Storz Endoscopy). Two sequential portals were placed using 6 mm threaded cannulas under laparoscopic guidance to enable a triangulated laparoscopic approach. A fourth portal was considered optional, depending on the intra-abdominal situation. Blunt, Babcock, and Kelly dissecting forceps (Clickline, Karl Storz Endoscopy) were used for tissue manipulation and dissection. A LigaSure™ Maryland tissue sealer/divider connected to a ForceTriad Energy platform (Medtronic Covidien Valleylab, Eindhoven, the Netherlands) was used for tissue dissection and transection.

If blood glucose levels were < 2.5 mmol/L, glucose 5% or 20% infusion was administered at 1 mg/kg/min rate, and blood glucose levels were measured every 15 min. If blood glucose was >3.0 mmol/L, no additional glucose was administered. During excision of the insulinoma, glucose administration was always discontinued to monitor intrinsic blood glucose level. If blood glucose would not increase to physiological or higher levels within 20 min after tumor excision, continued exploration for abnormal lymph nodes or liver metastasis and excision of any suspicious draining lymph node or liver lesion should be considered until blood glucose increases.

Excised tissues were send to the Veterinary Pathology Diagnostic Center, Department of Biomolecular Health Sciences, Faculty of Veterinary Medicine, Utrecht University, for histopathological examination.

## 3 Results

### 3.1 Surgery in clinical patients

Four canine patients with an insulinoma located in a pancreatic limb and staged negative for metastasis on triple-phase CECT received a partial pancreatectomy through lateral flank approach (3 x left, 1 x right) in sternal recumbency for excision of the insulinoma. All patients were pre-medicated with methadone (0.2 mg/kg IV) and midazolam (0.3 mg/kg IV). Induction of anesthesia was executed with propofol (1–2 mg/kg IV, repeated to effect) until intubation was feasible. After endotracheal intubation, anesthetic maintenance was provided by isoflurane in a mixture of oxygen in air (1:1). During anesthesia continuous rate infusion (CRI) of ketamine and sufentanyl were administered in all but one case. Case 4 received medetomidine (10 μg/kg IV) in conjunction with midazolam and propofol during induction. In this case, no CRIs were administered during the procedure. An additional administration of methadone was administered intraoperatively whenever deemed necessary. In all but one dog (case 4), a pre-operative dose of cefazoline (20 mg/kg IV) was administered which was repeated every 90 min intraoperatively.

Abdominal access was without difficulties in all cases. After routine exploration of the dorsolateral abdomen, the pancreas was approached. In the right-sided approach (case 1), the pancreas was in plain sight to the level of the corpus (Figure 2A). In this case, the insulinoma was located midway between the right pancreatic limb in the region of the accessory pancreatic duct with a relatively large part of pancreatic limb distal to it. To aid dissection of the limb out of the mesoduodenum, a fourth portal in the caudal abdominal wall was placed to enable a more parallel position of the tissue sealer. Excision of the distal pancreatic limb including the insulinoma was performed from distal to proximal, transecting the mesoduodenal attachment close to the pancreas, combined with blunt dissection more proximally, where the pancreas was closely associated with the duodenum. Because of the perceived proximity of the accessory pancreatic duct, transection of the pancreas was performed close to the tumor cranially with a cranial excision margin of <1 cm. For left sided insulinomas, the 3-portal left flank approach was started as described above. The distal part of the pancreatic limb was accessed by inspecting the omental region cranioventromedial to the left kidney (Figure 2B). Often, a small area of pancreatic tissue was visible, and traction on surrounding omentum uncovered a larger part of the pancreatic limb. Excision of the insulinoma always implied excision of the tumor mass en-bloc including the pancreatic limb distal to the tumor, with dissection of the pancreas starting from distal to proximal. The distal pancreatic limb was carefully manipulated by grabbing its surrounding omental attachments. The distal limb was carefully dissected out of the omentum by transectimg the omentum close to the pancreatic tissue using LigaSure™ while monitoring that the splenic vein was not traumatized. Finally, pancreatic tissue proximal to the tumor was transected with LigaSure™, leaving a margin of normal appearing pancreas around the tumor. Excised tissues were retrieved with an extraction bag through a slightly enlarged portal. In three cases, laparoscopic biopsies of possible liver lesions, that were apparent during laparoscopic evaluation, were selected additionally, and in one case, a suspicious splenic lymph node adjacent to the tumor was removed en-bloc with the pancreatectomy. The areas of interest were checked for residual bleeding, and the abdomen was closed routinely. Directly after removal of the pancreatic neoplasia, blood glucose levels increased to upper normal range in all cases (Table 1).

**Table 1:** Overview of animals with an insulinoma treated by partial pancreatectomy through a laparoscopic flank approach in sternal-oblique recumbency.

Case	Signalment	Laparoscopic approach	Location insulinoma	Surgery Time	Pre-operative glycemia	Intra-operative glycemia, post-resection	Time to discharge from hospital	Peri-operative complications	Histology	Survival time post-surgery
1	Wire haired dachshund, 8 years, FC, 12.8 kg	Sternal-oblique, right flank; 4 portals	Right pancreatic lobe at level of accessory pancreatic duct	125 min.	3.2 mmol/L	5.0–7.0 mmol/L	2 days	Reduced appetite for 3 days	Multinodular endocrine carcinoma (ø2cm) with invasive growth in surrounding tissue and vascular invasion with clean-but-close surgical margin. No signs of metastasis in liver biopsy	1,211 days; alive at time of writing
2	German shepherd, 8 years, FC, 33.2 kg	Sternal-oblique, left flank; 3 portals	Distal left pancreatic lobe	94 min.	2.8 mmol/L	7.0–8.3 mmol/L	1.5 days	None	Multinodular malignant endocrine neoplasia with invasive growth and clean-but-close surgical margin	1,028 days; euthanized for reasons unrelated to insulinoma
3	Jack Russel terrier, 9 years, FC, 8 kg	Sternal-oblique, left flank; 3 portals	Distal left pancreatic lobe	66 min.	2.2 mmol/ L	5.2–6.2 mmol/L	1.5 days	None	Multinodular malignant endocrine neoplasia with vascular invasion and irregular ingrowth in surrounding tissue close to excision margin; no signs of metastasis in liver biopsy	673 days; euthanized because of insulinoma recurrence
4	Boxer, 7 years, FC, 29.8 kg	Sternal-oblique, left flank; 3 portals	Proximal left pancreatic lobe and splenic lymph node	99 min.	3.1 mmol/L	5.8–7.8 mmol/L	2.5 days	Reduced appetite and some abdominal discomfort for 1.5 days	Endocrine carcinoma with infiltration in surrounding pancreatic tissue with tumor-free excision margins; metastasis in local lymph node, no signs of metastasis in liver biopsy	564 days; alive at time of writing

FC, female castrated.

All cases recovered in the intensive care unit (ICU), with post-operative fluid-therapy of crystalloids and analgesia by methadone or buprenorfine, paracetamol, and ketamine and/or sufentanyl CRI if deemed necessary. Hospitalization times ranged from 1.5 to 2.5 days. Post-operative treatment in the home environment consisted of carprofen, tramadol, gabapentine, and/or paracetamol.

In all cases, histopathologic evaluation of pancreatic tumors resulted in a diagnosis of malignant endocrine tumor (or carcinoma), in light of the clinical picture further specified as insulinoma. Neoplasia-free margins were acquired in all cases, albeit with a minimal tumor-free resection margin in 3 cases. The dog, in which a lymphadenectomy was performed, had histopathological evidence of lymph node metastasis.

### 3.2 Case 1

A female castrated, wire-haired dachshund of 8 years old was initially presented for episodic seizures, generalized tremors, and exercise intolerance. Pre-anesthetic blood work for MRI of the cranium revealed a mild hypoglycemia of 3.9 mmol/L (reference 4.2–5.8 mmol/L). The MRI examination showed no abnormalities. After 1 month, glycemia was rechecked and measured at 3.8 mmol/L. Concomitant hyperinsulinemia was measured at 67.0 mIU/L (reference 11.6–29.0 mIU/L), confirming a diagnosis of insulinoma. On abdominal triple-phase CECT-scan after 1 month, a ø9 mm mass was observed midway in the right pancreatic lobe. No lymph node abnormalities were found. Awaiting a surgery appointment, diazoxide was started (4 mg/kg, twice daily, per oral). After 1 month, a partial pancreatectomy was performed through a right lateral flank approach in sternal recumbency as described above with placement of four portals. Macroscopically, the pancreatic lobe distal to the tumor appeared mildly hyperemic. During the pancreatectomy, the accessory pancreatic duct was most probably transected, based on intraoperative findings and anatomic location of the insulinoma (Figures 2A, 3A). Subsequently, laparoscopic cup-forcep biopsies were taken from a possible focal liver lesion with minor intraoperative bleeding, which was self-limiting. The size of the resected pancreatic tissue fragment was 7.6×1.5×2 cm. Glycemic levels were 3.2 mmol/L pre-operative, increased to 5.0–7.0 mmol/L intraoperatively after tumor resection, and increased further to 7.5–8.9 mmol/L during recovery in ICU. Total duration of the procedure was 125 min. During hospital stay, the appetite of the dogs was low, but small amount of food was ingested when fed forcefully. Analgesia was gradually tapered. After 2 days of surgery, the dog was clinically stable and normoglycemic, allowing discharge from the ICU. Appetite recovered completely in the home setting. Histopathologic examination confirmed a ø2cm invasive malignant proliferation of the endocrine pancreas with minimal tumor-free margins and signs of vascular invasion. Additionally, signs of pancreatitis were observed in the remaining pancreatic tissue. The liver biopsy showed signs of reactive hepatitis, without signs of metastasis. Short-term recovery was excellent, and no complications were noted. At a 7-month post-surgery follow-up call with the owners, the dog was clinically unremarkable. After 11 months, the dog presented symptoms which were unrelated to the initial consultation, namely, diarrhea, vomiting, and anorexia. During the consultation, the dog was normoglycemic (4.8 mmol/L), no further examinations were performed, and the dog was discharged with a symptomatic treatment. The latest follow-up call was performed after 1,211 days of surgery. Apart from several subcutaneous mass lesions in the axillary and inguinal region, the dog seemed clinically healthy. At the time of owner contact, the survival time of this patient was 1,232 days after diagnosis, and no recurrence of clinical signs was noted.

### 3.3 Case 2

Case 2 was a female castrated, German Shepherd of 8 years old with clinical signs of weakness and tremors. Hypoglycemia (2.6 mmol/L) raised the suspicion of insulinoma, although the blood insulin level of 10mIU/L was just below the normal reference range (11.6–29.0). Triple phase abdominal CECT scan revealed a ø15-mm nodule in the distal left pancreatic lobe, which, in conjunction with the clinical signs and hypoglycemia, made a diagnosis of insulinoma highly likely. After 1 week, a partial pancreatectomy through a 3-portal left laparoscopic flank approach in sternal recumbency was performed as described above. Access to and excision of the insulinoma was straight-forward (Figure 3B), without complications and intra-abdominal exploration, which showed no further abnormalities. The left pancreatic lobe was transected 1 cm proximal of the visible tumor. The resected pancreatic fragment measured at 5.1×3.5×1.5 cm. Glycemic levels were 2.8 mmol/L pre-operative, increased to 7.0–8.3 mmol/L intraoperative after tumor resection, and remained between 6.5 and 9.3 mmol/L during recovery in ICU. Total duration of the procedure was 94 min. During recovery, appetite gradually recovered, and analgesia was tapered. After surgery, the dog was clinically stable and normoglycemic and was discharged from the ICU after 1.5 days. Histopathologic examination of the mass with a diameter of approximately 1.5 cm confirmed an invasive growing malignant proliferation of the endocrine pancreas with minimal tumor-free margins. No clinical abnormalities were noted during a control visit at our referral center after 1 month, and neither recurrent clinical signs nor hypoglycemia, which was measured at home by the owners, were present thereafter as monitored through regular follow-up telephone calls. The dog was euthanized after 1,028 days of surgery due to pulmonary metastasis, possibly related to a splenic mass of unknown origin which was removed before 6 months. Survival time from diagnosis until euthanasia was 1,035 days, without recurrence of hypoglycemia in the mentioned time-period.

### 3.4 Case 3

A 9-year-old female castrated, Jack Russel Terrier was referred with a 2-month history of seizures and weakness, possibly related to prolonged intervals between meals. During diagnostics performed by the referring veterinary practice blood examination revealed hypoglycemia (2.8 mmol/L) and an abdominal ultrasound showed a pancreatic nodule in the left pancreatic lobe. Upon referral, hypoglycemia was also noted (2.3 mmol/L) while blood insulin level was within the normal range (18.0 mIU/L). A triple-phase CECT scan confirmed the presence of a ø14 mm mass-like structure in the distal left pancreatic lobe without signs of concurring metastasis. During 1.5 weeks prior to surgery the dog’s diet was adjusted (increased feeding frequency of lower quantities) as supportive measure. A 3-portal left flank approach in sternal recumbency easily revealed the mass in the distal left pancreatic lobe (Figure 3C), which was excised using LigaSure™ without damaging the closely associated splenic vein. A cup-forcep liver biopsy was taken of a small pale colored nodule of 2 mm in diameter in the left lateral liver lobe. Resected pancreatic tissue measured at 2.1×1.5x1cm. Glycemic levels were 2.2 mmol/L pre-operative, increased to 5.2–6.2 mmol/L intraoperative, and ranged between 5.5 and 10.5 mmol/L during recovery. Surgery duration was 66 min. During recovery, appetite gradually recovered, and analgesia was tapered quickly to less potent oral analgesics, and the dog was discharged from ICU after 1.5 days of surgery. Histopathologic examination of the mass with a diameter of approximately 1.5 cm confirmed malignant proliferation of the endocrine pancreas with invasive growth in surrounding fatty tissue and suspected invasion of blood vessels. The liver biopsy histopathology was consistent with steroid-induced hepatopathy without signs of metastasis. Short-term outcome was excellent, with increased activity and subsided seizures. After 3 months of surgery, a follow-up call was performed, and the dog was clinically unremarkable. The dog was presented, however, 246 days after surgery at our institution with recurrence of clinical signs of weakness, especially present after a prolonged interval between meals. Hypoglycemia (2.7 mmol/L) in conjunction with hyperinsulinemia (45.0 mIU/L) was noted. Following the initial blood examination, an abdominal ultrasound was performed, without any signs of local recurrence and/or metastasis. The owners did not choose to repeat CT staging. Diazoxide (5 mg/kg, twice daily, PO) was started, and meals were more frequently offered in order to manage hypoglycemia. Due to progressing disease, likely related to the progression of insulinoma and shown as generalized weakness and gastrointestinal signs, the dog was euthanized 690 days after diagnosis.

### 3.5 Case 4

A 7-year-old female castrated Boxer showed episodic weakness and imbalance in combination with tremors of the right hind leg. After performing multiple blood examinations, insulinoma was diagnosed due to hypoglycemia (2.2 mmol/L) and concomitant hyperinsulinemia (49mIU/L). The dog was referred to our hospital after 2 weeks. At this time, the dog showed no abnormalities in a general clinical examination. An ultrasound of the abdomen was executed, which raised suspicion of a mass in the left pancreas. Prednisolone (0.25 mg/kg twice daily PO) and diazoxide (5 mg/kg daily divided in two doses PO) were started, awaiting further diagnostics. Triple-phase abdominal CECT scan was performed 1.5 weeks later, showing a ø2cm pancreatic nodule situated proximally in the left pancreatic limb close to the corpus. No signs of local or distant metastasis were noted. After 2 weeks, a partial pancreatectomy was performed through a left sided 3-portal laparoscopic flank approach in sternal recumbency. Upon inspection of the abdomen, the distal part of the left pancreatic limb was localized and freed from the surrounding omental attachments. The distal pancreatic limb was notably abnormal with firm consistency, hyperemic, and with dark pink color up until the tumor was noted in the proximal left pancreatic limb. Dissection of the distal pancreatic lobe from its omental attachments was complicated due to the relatively proximal tumor location and ample fat deposition in the omentum. Care was taken to dissect close to the pancreatic tissue to prevent trauma to splenic blood vessels and other structures in closer proximity of the pancreatic corpus. Increased traction on the lobe was necessary to reach and dissect the tumor, which was free from its surroundings. During dissection, an abnormal lymph node in close conjunction with the left pancreatic lobe was observed just proximal to the pancreatic tumor, suspected to be a metastatic splenic lymph node. This lymph node was excised en-bloc with the partial pancreatectomy of the affected limb. A biopsy of the liver was performed in an area, where some small pale-colored non-protruding lesions were visible. The size of the pancreatic excision was 8×2.5x1cm. Mild self-limiting intraoperative bleeding was noted from the pancreatectomy and liver biopsy site. During the procedure, glycemic levels rose from 3.1 mmol/L pre-operative to 5.8–7.8 mmol/L intraoperative after excision of the neoplasia. Total duration of the procedure was 99 min. During post-operative hospitalization in the intensive care unit glucose levels were hypo-to normoglycemic in between 3.8–4.8 mmol/L without adjuvant treatment of the hypoglycemia, except that the prednisolone therapy which the dog received prior to surgery was continued in a tapering dose scheme over the course of 2 weeks to be discontinued after this treatment schedule. During the hospital stay, the dog remained moderately painful in the abdomen initially and seemed nauseous intermittently. These symptoms were treated with add-on analgesics as described above and symptomatically with maropitant (1 mg/kg once daily IV), metoclopramide (1 mg/kg/day IV), and pantoprazole (1 mg/kg once daily IV), respectively. Appetite gradually recovered, and analgesia was tapered, and after 2.5 days of surgery, the dog was clinically stable and normoglycemic and was discharged from ICU. At home, treatment was briefly continued with oral analgesics, maropitant during 4 days, and the remaining tapering dose schedule of prednisolone. Histopathologic examination confirmed a ø2cm invasively growing malignant proliferation (carcinoma) of the endocrine pancreas, excised with tumor-free margins. However, in the adjacent lymph node, multiple nests of comparable neoplastic cells were noted. The liver biopsy showed signs of steroid-induced hepatopathy, without any signs of metastasis. Additionally, tumor Ki67 index was determined to be 5.8. At a 3-week post-operative control visit, the dog was completely recovered from the surgery, had no clinical abnormalities, and was normoglycemic (7.2 mmol/L). Follow-up calls with the owners were performed until 564 days after surgery. The animal was still clinically well and remained normoglycemic, as checked routinely by the owners. As yet, survival time, without recurrence, is 599 days after diagnosis for this patient.

## 4 Discussion

In this case series, a novel laparoscopic flank approach in sternal recumbency for partial pancreatectomy was successfully performed in 4 canine insulinoma patients. Patients were positioned in sternal recumbency, up to 30^o^ oblique, and were approached through either the right or left flank. No intraoperative complications occurred, and conversion to an open approach was not necessary. Post-operatively, all dogs recovered quickly and were discharged within 2.5 days. Overall, this minimally invasive approach provided good access to both pancreatic lobes and was well tolerated by patients.

The cases presented in this report were all female. This finding contrasted with a study of Del Busto et al., where the majority were male (14). Clinical signs upon presentation varied slightly from patient to patient, but all were showing episodic weakness, seizures, and/or tremors. A recent report on dogs with insulinomas showed that the majority of patients were displaying these clinical signs as well. Additionally, the episodic manner of occurring seems to be characteristic for this condition (3). The present patient group is, however, of small quantity, and no generalized conclusions can be formed.

A diagnosis of insulinoma was made based on a hypoglycemia with concurrent normo-or hyperinsulinemia in most cases (4). In one case, blood insulin level (10 mIU/L) was just below the reference value of the diagnostic lab used (11.6–29.0 mIU/L). However, in a normal dog with hypoglycemia, insulin levels are expected to be much lower. Therefore, with the combination of clinical signs, repeated measurements of hypoglycemia (2.6 mmol/L), and an insulin level of 10 mIU/L, a diagnosis of insulinoma was considered highly likely. The additional finding of a pancreatic nodule on CT evaluation was considered diagnostic for insulinoma, unless proven otherwise by histopathology. In fact, 10 mIU/L insulin level is considered a cutoff value in diagnosis of insulinoma in the face of glucose levels <3.5 mmol/L (4). However, reference range for normoinsulinemia may also vary between laboratories. Further diagnostic work-up consisted of the current standard for insulinoma staging and triple phase CT-angiography. Buishand et al. found this method to have the highest sensitivity (96%) in detecting insulinomas. However, defining the exact anatomic location of the tumor and prediction of possible metastasis was less reliable and was only accurate in 54 and 67% of the cases, respectively (11). Since the laparoscopic flank approach described above is considered only suitable for peripheral insulinomas in one specific pancreatic lobe, case selection is important. Patients with neoplastic lesions that seem ill-defined, affect the pancreatic body, or show signs of intra-abdominal metastases based on CECTangiography might not be appropriate candidates for laparoscopic removal.

In the present cases, all masses were situated in the pancreatic lobes, facilitating pancreatectomies of the respective parts. In one case, the neoplasia in the left pancreatic lobe was found relatively close to the pancreatic body, leading to a more challenging dissection. Overall, with traction and dissection close to the pancreas, the distal, two-thirds of the left lobe, is accessible through the left flank approach. Neoplasia, more proximal in the left lobe, is considered more difficult to reach through a left flank approach. In general, insulinomas in the pancreatic corpus and proximal right lobe are challenging because of the high-risk location. These sites only permit local marginal excision in case of small tumors, not reaching the area of central duct system, and intraoperative evaluation of the situation may be difficult using a laparoscopic approach because of the more limited maneuverability and lack of manual palpation.

In the author’s opinion, surgical staging, and thus assessing and removing lymph nodes potentially involved with insulinoma disease but not abnormal on CECT evaluation is very challenging through a laparoscopic approach, especially considering the numerous (up to 16) lymph nodes draining the pancreas and potentially containing metastatic disease (24). Proper case selection is therefore of importance, and cases with a neoplasia close to or in the pancreatic corpus or suspected metastasis in multiple loco-regional lymph nodes might benefit from an open approach.

Intraoperative monitoring of blood glucose levels to detect an increase to (supra) physiologic level after insulinoma excision is considered an effective means to estimate if measurable residual disease is present (25, 26), especially in the absence of visual abnormal or suspicious lymph nodes, or hepatic changes on CECT. All cases in the present report responded excellent intra-operatively, with increasing glycemia directly post insulinoma excision, thus preventing the need of additional lymph node excisions. This is further supported by the long-term follow-up in these cases. However, one should always be prepared to proceed with lymph node evaluation, and excision, in case glucose levels do not increase to physiologic level after excision of the primary insulinoma. Multiple lymph nodes may be accessible laparoscopically, especially from the right sided flank approach (22). In case the laparoscopic approach does not permit sufficient lymph node access or hypoglycemia persists, converting to an open surgical approach may be indicated for a more thorough and hands-on exploration. For future cases, intraoperative sentinel lymph node visualization, such as with near infrared fluorescence techniques, may improve intraoperative lymph node detection, either through celiotomy or laparoscopic approach.

The current sternal recumbency was previously described for adrenal surgery (21) and, in the current study, provides very good surgical access to large parts of the pancreas. Overall, the surgical access to the pancreatic lobes was considered very good and, subjectively, considerably improved compared with a ventral laparoscopic approach in dorsal or dorsal-oblique recumbency. For right sided pancreatectomy, the sternal recumbency prevents the need for constant manipulation and retraction of the duodenum and provides a direct view and access to the lobe. In author’s experience, access to the left pancreatic lobe is even more cumbersome in dorsal recumbency, with the need to manipulate and retract stomach, large parts of omentum, spleen, and intestines. In the left-sided flank approach in sternal recumbency, access to the left pancreatic lobe is considered much more straight-forward, the pancreatic limb being presented hanging in the dorsal omental leaf, the remaining omentum, stomach, spleen, and intestines passively moving out of the field of interest. In one case, more caudally located portal was placed to provide a better angle of approach (i.e., a more parallel orientation) with the LigaSure™ for dissecting the pancreatic lobe out of the mesoduodenum. Placement of an extra portal should always be considered in advanced laparoscopic procedures if the angle of approach is insufficient or extra retraction of tissues is needed. Surgery duration was comparable to a ventral laparoscopic approach (66–125 min versus 85 min) (20).

Excised tumors were histologically defined as malignant endocrine tumors or endocrine carcinomas and in conjunction with the clinical picture, further specified as insulinoma. The tumors displayed local invasive growth and signs of vascular invasion. Ki67 index was determined only in case 4, which resulted in a value (5.8) higher than the cutoff value (2.5) as defined by Buishand et al., indicating a worse prognosis (27). The same case also had metastasis present in a local lymph node. In the remaining cases, Ki67 index was not determined, and no lymph nodes were sampled, all appearing normal and small on CECT. The reason that a metastatic lymph node was missed on CECT in case 4 was probably due to the fact that this only mildly enlarged, but abnormal lymph node was closely associated with the primary tumor and was therefore not distinguishable as a separate nodule, as insulinoma often show a heterogeneous contrast enhancement on CECT (11).

Short-term outcome seemed to be excellent with a brief hospital stay and quick recovery at home. Diabetes mellitus and pancreatitis were not observed as post-operative complications. Glycemia was intermittently high in some of our patients during their hospital stay but never consistently. One dog had hypo-to anorexia while hospitalized, which may have been a result of mild local pancreatitis after transection of its accessory pancreatic duct. On the other hand, the reduced appetite may also have been linked to either anxiousness or side effect of (opioid) medication directly post-operative, especially considering that the dog improved within 2 days at home on oral analgesics. Long-term outcomes in comparison with other studies on insulinoma involving pancreatectomy seem to be very good. Overall, survival times after surgery ranged between 564 and 1,211 days, and only one dog had clinical recurrence after 229 days. The dog with relapse of clinical signs was managed medically with diazoxide and reached a survival time of 673 days. Although case 4 had apparent local lymph node metastasis and a Ki67 index above the reference value, the dog was still alive at the time of writing, 18 months later, without any signs of recurrence. One other case was euthanized due to splenic hemangiosarcoma with concurrent lung metastasis as assessed by the referring veterinarian. Its survival time was 1,232 days. However, it cannot be ruled out if this metastasis was indeed linked to the hemangiosarcoma or may have been metastasis of the insulinoma. However, blood glucose levels were monitored by the owners of this dog and were within the normal range. The remaining case was alive at the time of writing, without any clinical abnormalities related to the neoplastic condition, thus having a current survival time of 1,035 days. Survival times were higher than a recent report for an open approach, wherein MST of 378 days was found (14). However, our case series probably consisted of dogs with less severe disease, being without apparent metastasis on CECT, with only one patient with stage 2 disease as detected during surgery (case 4). Furthermore, survival times of dogs that show resolution of hypoglycemia after insulinoma removal have a better prognosis. All four cases described showed resolution of hypoglycemia, and survival times were comparable with dogs that showed resolution of hypoglycemia after surgery (746 days) in a report of 49 cases (5).

As a general conclusion, we state that a laparoscopic flank approach in sternal recumbency provides good surgical access to perform distal pancreatectomies of the right and left pancreatic lobes. In the cases described above, no significant peri-operative complications occurred, and recovery was rapid. Furthermore, with proper staging and case selection, laparoscopic treatment of patients with insulinoma is highly feasible with long-term disease-free survival possible. In addition, intraoperative monitoring of blood glucose levels seems an effective measure of disease burden. Consequently, insufficient blood glucose increase after primary insulinoma excision probably warrants additional surgical staging and measures to further detect and reduce tumor load to improve outcome. This may include conversion to an open approach because of the difficulties involved with complete surgical staging of insulinoma disease.

## Data availability statement

The original contributions presented in the study are included in the article/Supplementary material, further inquiries can be directed to the corresponding author.

## Ethics statement

Ethical review and approval was not required for the animal study because all data reported in this manuscript was based on client-owned dogs that were treated in our hospital for their pancreatic tumor; procedures or diagnostic tests outside of the regular regimen were not performed. Written informed consent was obtained from the owners for the participation of their animals in this study. Written informed consent was obtained from the individual(s) for the publication of any potentially identifiable images or data included in this article.

## Author contributions

JK: Writing – original draft, Writing – review & editing. SN: Writing – review & editing.
